# Evaluation of Alpha-Thalassemia Mutations in Cases with Hypochromic Microcytic Anemia: The İstanbul Perspective

**DOI:** 10.4274/tjh.2014.0204

**Published:** 2015-12-03

**Authors:** Zeynep Karakaş, Begüm Koç, Sonay Temurhan, Tuğba Elgün, Serap Karaman, Gamze Asker, Genco Gençay, Çetin Timur, Zeynep Yıldız Yıldırmak, Tiraje Celkan, Ömer Devecioğlu, Filiz Aydın

**Affiliations:** 1 İstanbul University İstanbul Faculty of Medicine, Department of Pediatric Hematology-Oncology, İstanbul, Turkey; 2 İstanbul University İstanbul Faculty of Medicine, Department of Medical Biology, İstanbul, Turkey; 3 İstanbul University İstanbul Faculty of Medicine, Department of Medical Biology, İstanbul, Turkey; 4 Göztepe Education and Research Hospital, Clinic of Pediatric Hematology, İstanbul, Turkey; 5 Şişli Etfal Education and Research Hospital, Clinic of Pediatric Hematology, İstanbul, Turkey; 6 İstanbul University Cerrahpaşa Faculty of Medicine, Department of Pediatric Hematology-Oncology, İstanbul, Turkey

**Keywords:** Anemia, Alpha thalassemia, Hb Adana, Hb Icaria, Hb Koya Dora, mutation, Thalassemia

## Abstract

**Objective:**

Alpha thalassemia syndromes are caused by mutations on one or more of the four α-globin genes. Mutations could be either more commonly deletional or non-deletional. As some deletions (3.7 and 4.2) cause α+-thalassemia, some cause (-20.5, MED, THAI, FIL) α0 -thalassemia. The aim of this study was to determine alpha thalassemia mutations in patients with unsolved hypochromic microcytic anemia and to evaluate types of mutations.

**Material and Methods:**

Two hundred six patients with hypochromic microcytic anemia were evaluated for alpha thalassemia. A venous blood sample of 2 mL was drawn from each patient for DNA isolation. The samples were investigated for α-thalassemia mutations by using the Vienna Lab α-Globlin StripAssay TM commercial kit.

**Results:**

Fourteen different mutations were determined in 95 (46.1%) patients. The most common mutation was the 3.7 single gene deletion and was found in 37 patients (n=37/95, 39%). Others common mutations were the 20.5 kb double gene deletion (n=20 patients, 21%), MED double gene deletion (n=17 patients, 17.9%), α2 IVS1 (n=10 patients, 10.5%), α2 cd142 Hb Koya Dora (n=6 patients, 6.3%), α2 polyA1 (Saudi type) (n=6 patients, 6.3%), 4.2 single gene deletion (n=4 patients, 4.2%), α1 cd14 (n=2 patients, 2.1%), and -FIL mutation (n=2 patients 2.1%), respectively. Hb Adana, Hb Icaria, α2 init cd and α2 polyA2 (Turkish type) were found in 1% of the patients (n=1). Seven patients (7.4%) had α-thalassemia triplication. In our study, three mutations (Hb Icaria, α1 cd14, α2 init.cd) were determined firstly in Turkey. Seven mutations (-SEA, -THAI, Hb Constant Spring, α2 cd19, α2 cd59, α2 cd125, Hb Paksé) were not determined in this study.

**Conclusion:**

Alpha thalassemia should be considered in the differential diagnosis of hypochromic microcytic anemia especially in cases without iron deficiency and b-thalassemia carrier state. Genetic testing should be performed for the suspicious cases. We also recommend that a national database with all mutations in Turkey should be created to screen the alpha thalassemia cost-effectively.

## Abstract (tr)

**Amaç:**

Alfa talasemi sendromları, bir ya da daha fazla a-globin genindeki mutasyonlardan kaynaklanır. Mutasyonlar genelikle delesyonel olmakla birlikte non-delesyonel de olabilir. Bazı delesyonlar (3.7 ve 4.2) α+-talasemiye neden olurken bazıları da (-20.5, MED, THAI, FIL) α0-talasemiye yol açar. Bu çalışma ile İstanbul ilinde, diğer nedenlerle açıklanamayan hipokrom mikrositer anemili olgularda alfa talasemi mutasyonlarını belirlemeyi ve mutasyon tiplerini değerlendirmeyi amaçladık.

**Gereç ve Yöntemler:**

Bu çalışmada 206 hasta alfa talasemi için değerlendirmeye alındı. Her hastadan DNA izolasyonu için 2 ml venöz kan örneği alındı. Strip analiz kiti (ViennaLab Diagnostics GmbH, Austria) kullanılarak alfa talasemi mutasyonları araştırıldı.

**Bulgular:**

Doksan beş hastada (%46,1) 14 farklı mutasyon tespit edildi. En sık saptanan mutasyon 3.7 tek gen delesyonu idi (n=37 hasta, %39). Diğer mutasyonlar sıklık sırasına göre; 20,5 kb çift gen delesyonu (n=20, %21), MED çift gen delesyonu (n=17, %17,9), α2 IVS1 (n=10, %10,5), α2 poly-A1 (Suudi tip) (n=6, %6,3), Hb Koya Dora (n=6, %6,3), 4.2 tek gen delesyonu (n=4, %4,2), FIL mutasyonu (n=2, %2,1) ve α1 cd 14 (n=2, %2,1) idi. Hb Adana (n=1), Hb Ikaria (n=1), α2 init cd (n=1) ve α2 poly-A2 (Türk tipi) (n=1) hastaların %1’inde saptandı. Yedi hasta alfa talasemi gen triplikasyonu (%7,4) taşıyordu. Çalışmamızda üç mutasyon (Hb Icaria, α1 cd14, α2 init.cd) Türkiye’de ilk kez tespit edildi. Yedi mutasyon ise (-SEA, -THAI, Hb Constant Spring, α2 cd19, α2 cd59, α2 cd125, Hb Paksé) hastalarımızda hiç saptanmadı.

**Sonuç:**

Alfa talasemi, hipokrom mikrositer anemilerin ayırıcı tanısında özellikle de demir eksikliği ve beta-talasemi taşıyıcılığının saptanmadığı durumlarda akla getirilmelidir. Şüpheli olgularda genetik açıdan mutasyon taraması yapılmalıdır. Alfa talasemi taramasını daha uygun maliyetle yapabilmek için Türkiye’de saptanan tüm alfa talasemi mutasyonlarının toplandığı ulusal bir veritabanı oluşturulmasını önermekteyiz.

## INTRODUCTION

Alpha thalassemia syndromes are inherited autosomal recessively and caused by defects on one or more of the 4 α-globin genes (αα/αα), leading to reduced or absent production of the alpha-globin polypeptide chains [1,2]. The α-globin gene mutations could be either the more common deletion (partial (α+) deletions or total (α0) deletions) or non-deletional types. There are reported to be more than 40 deletion mutations in various studies [2,3,4]. The most common alpha-thalassemia mutations in the world are the 3.7 single-gene deletions. While α+-thalassemia is caused by single-gene deletions (such as 3.7 and 4.2), α0-thalassemia is caused by double-gene deletions (such as -20.5, SEA, MED, THAI, and FIL). Three-gene deletions (α+ with α0-thalassemia) or a combination of two-gene deletions with a non-deletion mutation cause HbH disease. If there are deletion mutations on 4 α-genes, Hb Bart’s hydrops fetalis develops [5,6]. These large deletions have particularly severe phenotypes. On the other hand, non-deletion mutations result in structurally abnormal and instable hemoglobin variants such as Hb Constant Spring, which is the most common, and Hb αTSaudiα, polyA α2, Hb Koya Dora, and Hb Quong Sze [4,7]. Non-deletion mutations may reduce α-globin chain synthesis more severely than most of the deletion mutations [1]. More than 70 non-deletion mutations have been reported [8].

The clinical course of alpha thalassemia is correlated with the number of affected α-globin genes. There are 4 clinical definitions of α-thalassemia syndromes: 1) silent carrier, defined as heterozygous α+-thalassemia (-α/αα) with mostly normal hemoglobin or mild hypochromic anemia; 2) α-thalassemia trait, defined as heterozygous α0-thalassemia (--/αα) or homozygous α+-thalassemia (-α/-α) with mild anemia; 3) HbH disease, defined as compound heterozygous α+/α0-thalassemia with 3 inactive α-genes (--/-α) with moderate hemolytic anemia; and 4) Hb Bart’s, defined as homozygous α0-thalassemia (--/--) with hydrops fetalis. Silent carriers and those with α-thalassemia trait are generally clinically asymptomatic and do not need any treatment. Patients with HbH disease usually have moderate anemia with hepatosplenomegaly; some of them need periodic blood transfusion and folic acid supplementation. Hb Bart’s causes hydrops fetalis prenatally and is fatal if not treated with intrauterine blood transfusions [2,3,8].

The aim of this study was to determine alpha-thalassemia mutations in patients with unsolved hypochromic microcytic anemia and to evaluate the types of mutations.

## MATERIALS AND METHODS

Two hundred six individuals either having hypochromic microcytic anemia or being parents and/or siblings of a patient with HbH disease were referred to our institution for screening of alpha thalassemia mutations in İstanbul. A venous blood sample of 2 mL was drawn from each patient into the EDTA tubes for DNA isolation. In vitro amplification was made with polymerase chain reaction (PCR) multiplex method using Biotin marked primers belonging to alpha globin encoding gene zones. Products of the amplification process were investigated for mutations of the alpha globulin genes using the Vienna Lab α-Globlin Strip Assay TM commercial kit including 21 alpha thalassemia mutations. These mutations are shown in Table 1.

## RESULTS

Ninety-five patients (46.1%) with alpha thalassemia mutations were identified. The patients were aged from 1 to 46 years; 52 of those were male and 43 were female. Deletion mutations were detected in 69.3% of the patients whereas non-deletion mutations in 30.6%. The most common mutation was the 3.7 single gene deletion and was found in 37 patients (39%). Others common mutations were the 20.5 kb double gene deletion (n=20 patients, 21%), MED double gene deletion (n=17 patients, 17.9%), α2 IVS1 (n=10 patients, 10.5%), α2 cd142 Hb Koya Dora (n=6 patients, 6.3%), α2 polyA1 (Saudi type) (n=6 patients, 6.3%), 4.2 single gene deletion (n=4 patients, 4.2%), α1 cd14 (n=2 patients, 2.1%), and -FIL mutation (n=2 patients 2.1%), respectively. Hb Adana, Hb Icaria, α2 init cd and α2 polyA2 (Turkish type) were found in 1% of the patients (n=1). Seven patients (7.4%) had α-thalassemia triplication (Table 2). Some deletions (-SEA, -THAI) and some mutations (α2 cd19, α2 cd59, α2 cd125, Hb Paksé and Hb Constant Spring) were not determined in this study (Table 1).

Fourteen distinct alpha thalassemia mutations were detected in 95 patients. In the total of 190 alleles, the most common mutation was the 3.7 single gene deletion (n=37 alleles, 19.5%). The allele frequencies of the other mutations were: 20.5 kb double gene deletion (n=20 alleles, 10.5%), MED double gene deletion (n=17 alleles, 8.9%), α2 IVS1 (n=10 alleles, 5.2%), α2 polyA1 (Saudi type) (n=7 alleles, 3.7%), alpha triplication (n=7 alleles, 3.7%), Hb Koya Dora (n=6 alleles, 3.1%), 4.2 single gene deletion(n=4 alleles, 2.1%), α1 cd14 (n=2 alleles, 1%), and -FIL mutation (n=2 alleles, 1%), respectively (Table 3). The allele frequencies of Hb Adana, Hb Icaria, α2 init cd and α2 polyA2 (Turkish type) were found to be 0.5% (Table 3). Three mutations (Hb Icaria, α1 cd14, α2 init.cd) were detected for the first time in Turkey.

The genetic results of the patients showed that 28 patients (29.4%) were silent carriers, 45 patients (47.3%) had alpha thalassemia trait, and 15 patients (15.8 %) had Hemoglobin H disease (Table 2). Seven patients with alpha triplication were not grouped phenotypically.

## DISCUSSION

According to reports from the World Health Organization, at least 20% of the world’s population is alpha-thalassemia carrier [9]. The geographic distribution of α-thalassemia mutations is especially concentrated in the Mediterranean and Middle Eastern regions, where up to 40% of people are carriers [4,10]. Turkey also has a high alpha-thalassemia frequency because of its geographic position.

The first publication on alpha-thalassemia from Turkey was that of Özsoylu and Malik, who studied alpha-thalassemia by column chromatography in 1982 [11]. The first screening study of alpha-thalassemia by sensitive DNA method (gene mapping) was published in 1989 [12]. The frequency of alpha-thalassemia was detected at 3.6%, while the frequency of alpha-gene triplication was also 3.6%. Arcasoy reported that the frequency of alpha-thalassemia in Turkey was 0.25% [13]. Kılınç et al. studied the cord blood of newborns and reported the frequency of alpha-thalassemia carriers to be 2.9% in the Adana region in 1986, while Canatan et al. reported the frequency of alpha-thalassemia as 2.5%-6.5% in the Antalya region [14,15,16]. Guvenc et al. reported the incidence of α-thalassemia as 7.5% in selected patients in the Adana region [17]. The diagnosis of alpha-thalassemia is also important in patients with unsolved hypochromic microcytic anemia. We found a rate of alpha-thalassemia as high as 46.1% among selected patients with hypochromic microcytic anemia in İstanbul.

The types of alpha-thalassemia mutations are variable depending on geographic region. Although 21 mutations were screened, we found 14 different alpha-thalassemia mutations in patients who lived in İstanbul. The most common mutations were the 3.7 single-gene deletion, 20.5 double-gene deletion, MED double-gene deletion, α2 IVS-1, 3.7 gene triplication, Hb Koya Dora, α2 polyA1, 4.2 single-gene deletion, α1 cd 14, and FIL mutation, respectively. These mutations were seen in 95% of our patients, similar to reports from other parts of Turkey (Table 3).

According to the study by Guvenc et al., the rate of 3.7-kb deletion was extremely high (53.3%) in selected patients from Adana [17]. They also performed the largest study in Turkey with 3000 premarital couples and anemic patients, excluding those with iron deficiency, and they detected alpha-thalassemia mutations in 225 patients. They demonstrated 11 different genotypes; the 3.7 single-gene deletion and MED double-gene deletion were the most prevalent genotypes in their study.

Sütçü et al. reported that the most common alpha-thalassemia mutations were the MED double-gene deletion, 20.5-kb double gene deletion, 3.7 single-gene deletion, and α2IVS 1-5 nt, respectively, in the Isparta area in the south of Turkey [18]. Although they tested few patients and detected mutations in only 9 patients, their most common mutations were similar to those of other studies in Turkey.

Celik et al. demonstrated 9 distinct mutations and the frequencies of the mutations in Hatay [19]. They tested 330 individuals and detected mutations in 97 patients. Their inclusion criteria for the study were similar to ours. They reported that 3.7 single-gene deletions were the most common mutation at 43.81%. In addition, they reported FIL double-gene deletion for the first time in 2012 in Turkey. We also detected FIL mutation in 2 patients in İstanbul. In their study, deletion mutations were detected in 81.8% of the patients and non-deletion mutations in 18.2%, whereas deletion mutations were found in 69.3% and non-deletion mutations in 30.6% in our study. These findings are similar to those of other studies addressing alpha-thalassemia in the world.

HbH is the most common condition that arises from the deletions of 3 α-globin genes (--/-α) and rarely by the combination of deletion and non-deletion mutations affecting the α-globin genes. There are also published studies from Turkey about HbH disease [20,21,22,23]. Çürük reported mutations in 32 patients with HbH disease [20]. In that study, 20 patients with HbH had 3 alpha-gene deletions, while the remaining 12 cases were caused by the combination of alpha-gene deletion and point mutation. In our study, 15 patients were evaluated as having HbH disease; 9 of them had 3 α-globin gene deletions, 2 of them had non-deletion mutations, and the other cases were caused by the combination of deletion and non-deletion mutations, as shown in Table 2.

We found a very heterogeneous distribution of alpha-thalassemia mutations. This heterogeneity could be because İstanbul is the city in Turkey receiving the most migrants. We present the results of our study and other studies from Turkey in Table 3 [17,18,19,20,21,24,25]. In our study, we found 3 mutations that not been reported previously in Turkey. One-third of the mutations from the strip assay kit were not determined, similar to other studies from different parts of Turkey.

In our study, patients were identified as being silent carriers, having alpha-thalassemia traits, or having HbH disease on the basis of genetic mutations. For example, patients with single-gene deletion were defined as silent carriers. We determined clinical definitions for the patients according to their genetic mutations. Most silent carriers have normal hemoglobin levels in the general population. Our results showed that the silent carriers had mild hypochromic microcytic anemia because our study group included only patients with hypochromic microcytic anemia. Finally, silent carriers of alpha-thalassemia could be mostly normal or mildly anemic, as shown in the literature [2,3,8].

Screening for the 7 most common mutations, present in >95% of patients, is recommended in Canada [26]. The strip assay method for alpha-thalassemia genetic testing can be used effectively due to homogeneity of mutations in the public. It is also cost-effective for the most commonly seen mutations in patients with otherwise unexplained, long-standing, hypochromic microcytic anemia.

In conclusion, we found a high rate (46.1%) of alpha-thalassemia mutations among patients with long-standing hypochromic microcytic anemia. Alpha- thalassemia should be considered in the differential diagnosis of hypochromic microcytic anemia, especially in cases without iron deficiency and α-thalassemia carrier states. Genetic testing should be performed for these suspicious cases. Furthermore, we recommend that a national database including all mutations in Turkey should be created to screen alpha-thalassemia mutations cost-effectively.

## Figures and Tables

**Table 1:**
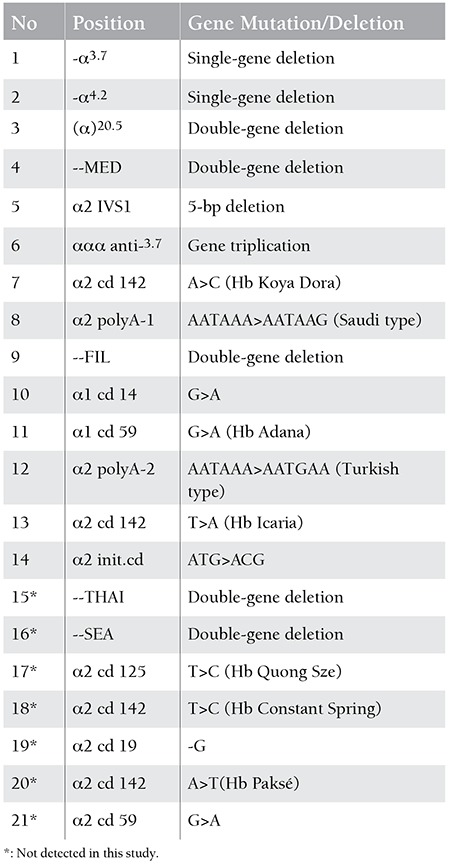
Positions of the 21 alpha-gene mutations in the strip assay kit.

**Table 2:**
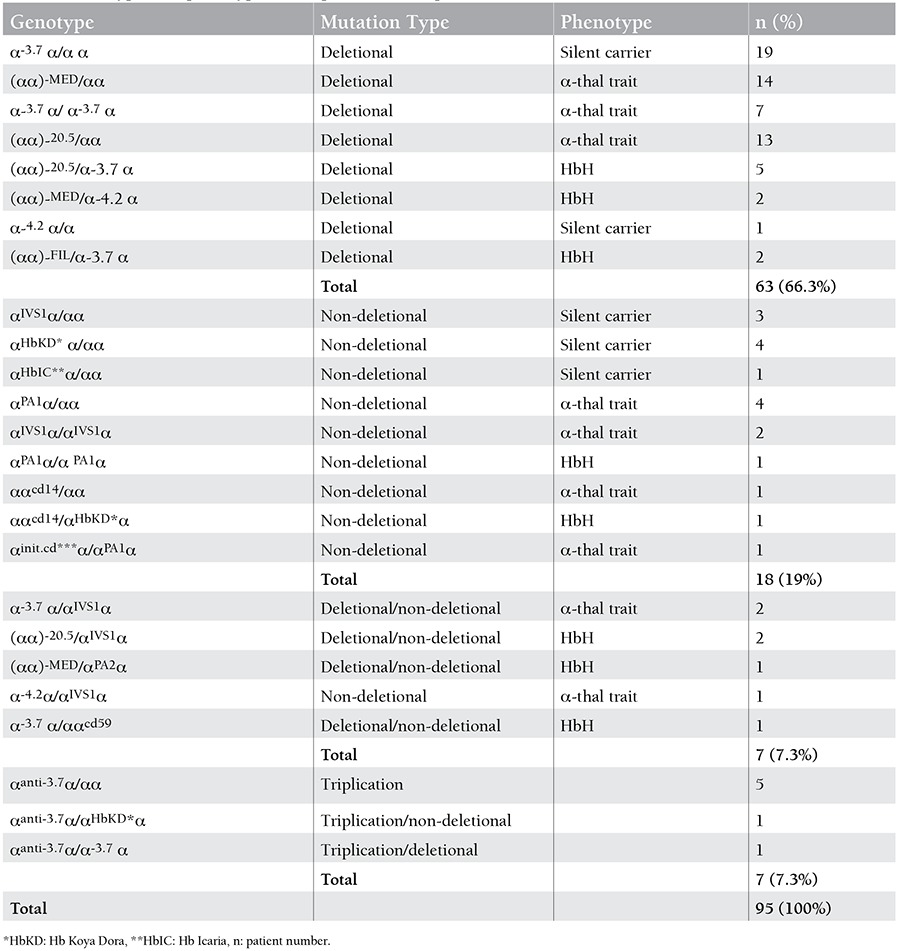
Genotypes and phenotypes of the patients with alpha-thalassemia.

**Table 3:**
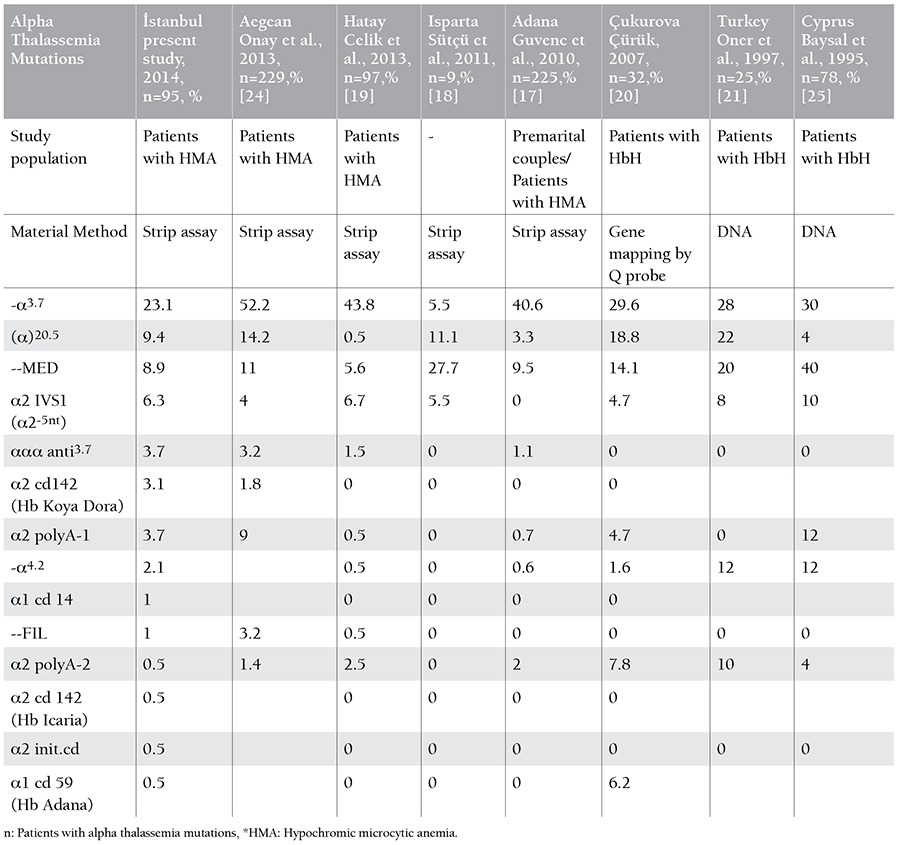
The allele frequencies of the alpha thalassemia mutations in different studies from Turkey.
